# Assessment of Information Sharing on Adverse Drug Reactions by Community Pharmacies with Other Medical Institutions

**DOI:** 10.3390/pharmacy10010025

**Published:** 2022-02-05

**Authors:** Daisuke Kikuchi, Taku Obara, Aoi Noda, Gen Oyanagi, Mami Ishikuro, Kouji Okada, Nariyasu Mano

**Affiliations:** 1 Department of Pharmacy, Tohoku Medical and Pharmaceutical University Hospital, 1-12-1 Fukumuro, Miyagino-ku, Sendai 983-8512, Japan; d.kikuchi@hosp.tohoku-mpu.ac.jp (D.K.); kokada@tohoku-mpu.ac.jp (K.O.); 2 Division of Molecular Epidemiology, Graduate School of Medicine, Tohoku University, 2-1 Seiryo-machi, Aoba-ku, Sendai 980-8573, Japan; a.noda@megabank.tohoku.ac.jp (A.N.); m_ishikuro@med.tohoku.ac.jp (M.I.); 3 Department of Preventive Medicine and Epidemiology, Tohoku University Tohoku Medical Megabank Organization, 2-1 Seiryo-machi, Aoba-ku, Sendai 980-8573, Japan; 4 Department of Pharmaceutical Sciences, Tohoku University Hospital, 1-1 Seiryo-machi, Aoba-ku, Sendai 980-8574, Japan; gen.oyanagi@hosp.tohoku.ac.jp (G.O.); mano@hosp.tohoku.ac.jp (N.M.); 5 Division of Clinical Pharmaceutics and Pharmacy Practice, Tohoku Medical and Pharmaceutical University, 1-12-1 Fukumuro, Miyagino-ku, Sendai 983-8512, Japan; 6 Graduate School of Pharmaceutical Sciences, Tohoku University, 1-1 Seiryo-machi, Aoba-ku, Sendai 980-8574, Japan

**Keywords:** adverse drug reactions, community pharmacy, providing information, questionnaire, information and communication technology

## Abstract

Widespread coordination and sharing of information regarding adverse drug reactions (ADRs) are important for drug safety assessment. However, the actual status of coordination and sharing of information on ADRs in community pharmacies remains unclear. Therefore, a survey was conducted at community pharmacies to analyze the status. In this cross-sectional study conducted from 31 March 2021 to 9 April 2021, a request letter with the uniform resource locator of the questionnaire form was sent to 302 community pharmacies affiliated with Tsuruha Holdings Inc., and the responses were obtained online. The response rate for the questionnaires was 80.8% (*n* = 244). In total, 20.9% of the community pharmacies provided information on patients’ ADRs to hospitals or clinics prescribing drugs. None of the community pharmacies provided patient ADR information to other community pharmacies. Of the community pharmacies, 98.8% felt that insufficient information was available to monitor ADRs from hospitals or clinics prescribing drugs. For example, the name of the disease (67.6%), considered to be the most common information, was insufficiently provided. Overall, the existing system for providing information on ADRs between community pharmacies and other medical institutions is insufficient and needs to be developed further.

## 1. Introduction

Adverse drug reactions (ADRs) are defined as any noxious and unintended response to a drug, occurring at doses commonly used for prophylaxis, diagnosis, or therapy of disease, or for the modification of physiological function [1]. Gandhi et al. reported an incidence of 27 ADRs per 100 patients in a prospective cohort study of outpatients at four adult primary care practices in Boston [2]. Overall, the probability of encountering ADRs in daily practice is very high.

Several studies have reported that many ADRs can be avoided or predicted [3,4]. Pirmohamed et al. conducted a prospective observational study of 18,820 patients at two large general hospitals in Merseyside, England, aged ≥ 16 years who required hospitalization for six months and reported ADRs in 6.5% of the patients [3]. Further, they found that 72% of the ADRs were avoidable [3]. Using a meta-analysis of the MEDLINE database, Beijer et al. reported that the prevalence of ADR-related hospitalizations was 4.1% in the young and 16.6% in the elderly [4]. They further suggested that 88% of ADR-related hospitalizations in the elderly and 24% in the young patients were preventable. Since some ADRs are avoidable and predictable, early detection of ADRs and sharing of relevant information among medical institutions are essential.

In recent years, the Ministry of Health, Labour and Welfare (MHLW) in Japan has contributed extensively to further improving the existing drug safety assessment system [5]. In addition, the medical care system has shifted from hospitals to communities—namely, outpatients at large hospitals were mainly referrals and general outpatient visits were being shifted to family doctors in the community [6]. Therefore, pharmacists affiliated with community pharmacies are expected to be more active in tasks related to drug safety assessment. ADR reporting by pharmacists is mandatory in the Pharmaceutical and Medical Device Act, and the collected ADRs are compiled into a Japanese Adverse Drug Event Report database, like EudraVigilance in Europe, contributing to the safety of drugs. Widespread coordination and sharing of information on ADRs are important for drug safety assessments. However, the actual status of this in community pharmacies remains unclear. Therefore, this study aimed to clarify the actual status of coordination and sharing of information on ADRs in community pharmacies.

## 2. Methods

### 2.1. Study Sample

This cross-sectional study was conducted in 302 community pharmacies belonging to Tsuruha Holdings Inc. in Japan. Community pharmacies belonging to Tsuruha Holdings Inc. are widely available throughout Japan, so they were chosen as the target community pharmacies for this study. From 31 March 2021 to 9 April 2021, a request letter with the uniform resource locator of the questionnaire form was sent to the community pharmacies affiliated with Tsuruha Holdings Inc., and responses were obtained online from pharmacists affiliated with each community pharmacy. One response per community pharmacy was used in this study.

### 2.2. Survey Items

The survey items in this study included the number of pharmacists belonging to each community pharmacy and the number of prescriptions per month, provision of information on ADRs to other medical institutions, tools used for sharing information, and insufficient data required for monitoring ADRs. The survey items were selective, although if the respondents chose “other” in the list of selected items, the contents were left as free text. The questionnaire survey in this study did not collect detailed information on patients’ ADRs. The survey was anonymous and did not include the names of the community pharmacies or any personal information.

### 2.3. Statistical Analysis

Categorical variables are presented as numbers and proportions. The Cochran–Armitage trend test adding exact trend was used for the basic characteristics of community pharmacies to provide information on ADRs to hospitals or clinics prescribing drugs. The chi-square test or Fisher’s exact probability test were used to analyze information that was insufficient for monitoring ADRs, irrespective of whether or not ADR information was provided from community pharmacies to the hospitals or clinics prescribing drugs. Statistical significance was set at *p* < 0.05. All statistical analyses were performed using SAS version 9.4 (SAS Institute Inc., Cary, NC, USA).

### 2.4. Ethical Approval

This study was approved by the Institutional Review Board of Tohoku University Tohoku Medical Megabank Organization (approval number: 2020-4-163). In this study, an explanatory document was sent to the eligible community pharmacies. Responses to the questionnaire constituted consent to participate in this study.

## 3. Results

The responses to this questionnaire are shown in Table 1. In this study, 38.1% of community pharmacies accepted 300–999 prescriptions per month, and 46.3% of community pharmacies had 2–4 pharmacists, which was the highest proportion among community pharmacies.

In this study, 20.9% (*n* = 51) of the community pharmacies provided information on patients’ ADRs to the hospitals or clinics prescribing drugs. Furthermore, none of the community pharmacies provided information on patients’ ADRs to other community pharmacies.

Among the community pharmacies (*n* = 51) that provided ADR information to hospitals or clinics, the names of suspected drugs and ADRs accounted for 86.3% of the ADR information provided. This was followed by the date of ADR onset (62.7%) and the date when the suspected drug was started or discontinued (52.9%). Similarly, the most common tools for providing information were other (e.g., telephone or fax) in 45.1%, followed by information forms for the proper use of drugs (23.5%) and printing or writing in the medication record book (15.7%).

Among the community pharmacies that responded to the questionnaire (*n* = 244), 98.8% of community pharmacies felt that they did not get enough information from hospitals or clinics prescribing drugs to monitor ADRs. The most common information that felt inadequate was the name of the disease (67.6%), followed by laboratory test results (63.5%) and physicians’ opinions and notes (60.7%).

The proportion of pharmacies that provided ADR information to the prescribing hospitals or clinics based on the basic characteristics of community pharmacies is shown in Table 2. There was a positive association between the number of prescriptions received per month and the provision of information provided on ADRs (*p* = 0.02) and between the number of pharmacists belonging to each community pharmacy and the provision of information provided on ADRs (*p* = 0.04).

The community pharmacies were divided into two groups based on whether or not they provided information on ADRs to prescribing hospitals or clinics. Table 3 shows the items for which community pharmacies felt that they did not have sufficient information to monitor ADRs in these two groups. Between these two groups, there was no statistically significant difference in the items on which community pharmacies felt that they were not getting sufficient information to monitor ADRs from prescribing hospitals or clinics.

## 4. Discussion

The MHLW stipulates that the maximum number of prescriptions that one pharmacist can fill is 40 per day. This is suggested by the fact that the number of prescriptions was low in the community pharmacies included in this study. The proportion of community pharmacies providing information on ADRs to the prescribing hospitals or clinics was only 20.9% in this study. Nagamitsu et al. reported that the action taken by nearly 90% of pharmacists in suspected ADRs is advising the patient to seek medical attention [7]. In addition, the most common reason for pharmacists’ reluctance to report to a physician was the uncertain causal relationship between the drug and ADR [7,8]. This suggests that uncertainty about the causal relationship between drugs and ADRs may be one of the factors that prevent pharmacists from reporting directly to physicians. Providing information about ADRs by community pharmacies to prescribing hospitals or clinics requires a system to obtain information regarding patients’ ADRs for community pharmacies. In some regions, community networks have been reported, wherein community pharmacies verify the information from hospitals or clinics [9,10]. In the near future, based on previous studies [9,10], it is necessary to build a network between community pharmacies and other medical institutions (hospitals, clinics, and other community pharmacies) to establish an information-sharing system for ADRs.

The MHLW has reported that an information-sharing system among healthcare professionals is essential for establishing a high-quality healthcare delivery system and that the use of information and communication technology (ICT) is an effective tool for information sharing [11]. However, in this study, traditional information-sharing tools such as telephone, fax, and writing in the medication record book were used more frequently compared to information-sharing tools such as ICT networks. The factors preventing the progression of ICT-based information-sharing tools are that ICT requires initial investment and running costs as recommended by the MHLW. Therefore, it is necessary to consider policies promoting the use of ICT in medical institutions along with the patients’ understanding of ICT.

More than 90% of the pharmacies in this study felt that they did not have enough information for monitoring ADRs from prescribing hospitals or clinics. In addition, they specifically needed information on the name of the disease and the results of the laboratory tests. Tokumaru et al. reported a survey on information feedback by community pharmacies concerning outpatient chemotherapy and found that approximately 80% of pharmacists at both hospitals and community pharmacies considered that the information provided by hospitals to community pharmacies was insufficient [12]. Thus, insufficient provision of relevant information is observed even for chemotherapeutic drugs, which are most prone to ADRs. Generally, prescriptions contain information such as the dosage and administration of the drug but not the name of the disease or the results of laboratory tests. Similar to previous studies, the results of this study indicate that for community pharmacies to identify ADRs, it is necessary for medical institutions to provide objective patient information such as disease names and laboratory test results.

In this study, community pharmacies with more pharmacists and more prescriptions constituted a higher percentage of community pharmacies providing information on ADRs to the prescribing hospitals or clinics. Further, there was no significant difference in the missing information for monitoring ADRs between community pharmacies that shared ADR information with the prescribing hospitals or clinics and those that did not. This suggests that the scale of the community pharmacies may be relevant to the provision of information on ADRs from community pharmacies to prescribing hospitals or clinics. However, other than the scale of community pharmacies, we could not clarify the detailed factors related to information sharing in ADRs.

This study has several limitations. Although the experience of a pharmacist is essential when interviewing patients about ADRs, only the number of community pharmacists was determined in this study. Tsuruha Holdings Inc. is the second-largest drugstore company in the industry in terms of sales in Japan. However, as a community pharmacy, it is a medium-sized company, so its representativeness of Japanese community pharmacies may not be able to be guaranteed.

## 5. Conclusions

Our findings indicate that the system for sharing information on ADRs between community pharmacies and other medical institutions remains insufficient. In addition, we found that regardless of whether community pharmacies provided ADR information to the hospitals or clinics prescribing drugs, there was no difference in the feeling that there was insufficient information to monitor ADRs.

## Figures and Tables

**Table 1:** Results of responses to the below questionnaire survey.

1. The number of prescriptions received per month (*n* = 244)		
	Less than 299, *n* (%)	79 (32.4)
	300–999, *n* (%)	93 (38.1)
	1000–1999, *n* (%)	51 (20.9)
	2000–2999, *n* (%)	16 (6.6)
	3000–3999, *n* (%)	5 (2.0)
	More than 4000, *n* (%)	0 (0.0)
2. The number of pharmacists belonging to each community pharmacy(*n* = 244)		
	1, *n* (%)	101 (41.4)
	2–4, *n* (%)	113 (46.3)
	5–9, *n* (%)	25 (10.2)
	10–19, *n* (%)	5 (2.0)
	More than 20, *n* (%)	0 (0.0)
3. Does your pharmacy provide information on ADRs to the hospital/clinic where the drug is administered or to other pharmacies? (*n* = 244)		
	We provide them to hospital/clinic where the drug is administered, *n* (%)	51 (20.9)
	We provide them to other pharmacies, *n* (%)	0 (0.0)
	We do not provide, *n* (%)	193 (79.1)
4. Information on ADRs provided from your pharmacy to the hospital or clinic where the drug is administered (multiple choices) (*n* = 51)		
	Name of the suspected drug, *n* (%)	44 (86.3)
	Name of ADR, *n* (%)	44 (86.3)
	The date of onset of the ADR, *n* (%)	32 (62.7)
	The date the suspected drug was started or discontinued, *n* (%)	27 (52.9)
	Dosage, *n* (%)	20 (39.2)
	Outcome, *n* (%)	7 (13.7)
	Other, *n* (%)	0 (0.0)
5. Tools used for providing information from your pharmacy to the hospital or clinic (*n* = 51)		
	Information forms for the proper use of drugs *, *n* (%)	12 (23.5)
	Printing or writing in the medication record book, *n* (%)	8 (15.7)
	Meeting, *n* (%)	5 (9.8)
	Printing and writing on the prescription, *n* (%)	2 (3.9)
	Systems installed in regional networks, *n* (%)	1 (2.0)
	Other (e.g., telephone or fax), *n* (%)	23 (45.1)
6. Information on ADRs provided from your pharmacy to other pharmacies (multiple choices) (*n* = 0)		
	Name of the suspected drug, *n* (%)	0 (0.0)
	Name of ADR, *n* (%)	0 (0.0)
	The date of onset of the ADR, *n* (%)	0 (0.0)
	The date the suspected drug was started or discontinued, *n* (%)	0 (0.0)
	Outcome, *n* (%)	0 (0.0)
	Dosage, *n* (%)	0 (0.0)
	Other, *n* (%)	0 (0.0)
7. Information that is not sufficient for monitoring ADRs at your pharmacy (multiple choices) (*n* = 244)		
	There is at least one piece of information that you feel is insufficient, *n* (%)	241 (98.8)
	The name of the disease, *n* (%)	165 (67.6)
	Clinical laboratory results, *n* (%)	155 (63.5)
	Physician’s opinion and notes, *n* (%)	148 (60.7)
	Oncotherapy administration regimen, *n* (%)	109 (44.7)
	Medication and history of ADRs during hospitalization, *n* (%)	106 (43.4)
	Height, weight, and body surface area, *n* (%)	30 (12.3)
	Other, *n* (%)	1 (0.4)

ADR; adverse drug reaction. * “Information forms for the proper use of drugs” is a document from the community pharmacy to the hospital or clinic to report the feedback of information recorded at the community pharmacy.

**Table 2:** The proportion of ADR-related information provided to the hospitals or clinics prescribing drugs based on the basic characteristics of community pharmacies.

	Community Pharmacies Providing Information on ADRs to the Hospitals or Clinics Prescribing Drugs, *n* (%)	*p*-Value
The number of prescriptions received per month		
Less than 299 (*n* = 79)	10 (12.7)	0.02
300–999 (*n* = 93)	18 (19.4)	
1000–1999 (*n* = 51)	19 (37.3)	
2000–2999 (*n* = 16)	2 (12.5)	
3000–3999 (*n* = 5)	2 (40.0)	
More than 4000 (*n* = 0)	0 (0.0)	
The number of pharmacists belonging to each community pharmacy		
1 (*n* = 101)	15 (14.9)	0.04
2–4 (*n* = 113)	27 (23.9)	
5–9 (*n* = 25)	7 (28.0)	
10–19 (*n* = 5)	2 (40.0)	
More than 20 (*n* = 0)	0 (0.0)	

ADR; adverse drug reaction. The Cochran–Armitage trend test adding exact trend was used for statistical analysis.

**Table 3:** Information that is insufficient for monitoring ADRs at your pharmacy (multiple choices).

	Information		*p*-Value
	Provided (*n* = 51)	Not Provided (*n* = 193)	
There is at least one piece of information that you feel is not sufficient, *n* (%)	50 (98.0)	191 (99.0)	0.40 ^b^
Results of laboratory tests, *n* (%)	34 (66.7)	121 (62.7)	0.60 ^a^
The name of the disease, *n* (%)	30 (58.8)	135 (69.9)	0.13 ^a^
Physician’s opinion and notes, *n* (%)	28 (54.9)	120 (62.2)	0.34 ^a^
Oncotherapy administration regimen, *n* (%)	20 (39.2)	89 (46.1)	0.38 ^a^
Medication and history of ADRs during hospitalization, *n* (%)	17 (33.3)	89 (46.1)	0.10 ^a^
Height, weight, and body surface area, *n* (%)	6 (11.8)	24 (12.4)	0.90 ^a^
Other, *n* (%)	1 (2.0)	0 (0.0)	0.21 ^b^

ADR; adverse drug reaction. ^a^ The chi-square test was used for statistical analysis. ^b^ Fisher’s exact probability test was used for statistical analysis.

## Data Availability

The research data are confidential.
